# Quantitative artifact reduction and pharmacologic paralysis improve detection of EEG epileptiform activity in critically ill patients

**DOI:** 10.1016/j.clinph.2022.11.007

**Published:** 2022-11-18

**Authors:** Catherine V. Kulick-Soper, Russell T. Shinohara, Colin A. Ellis, Taneeta M. Ganguly, Ramya Raghupathi, Jay S. Pathmanathan, Erin C. Conrad

**Affiliations:** a Department of Neurology, Perelman School of Medicine, University of Pennsylvania, Philadelphia, PA, USA; b Department of Biostatistics, Epidemiology, & Informatics, University of Pennsylvania, Philadelphia, PA, USA

**Keywords:** Neurocritical care, Continuous video EEG, EEG interpretation, Myoclonus

## Abstract

**Objective:**

Epileptiform activity is common in critically ill patients, but movement-related artifacts—including electromyography (EMG) and myoclonus—can obscure EEG, limiting detection of epileptiform activity. We sought to determine the ability of pharmacologic paralysis and quantitative artifact reduction (AR) to improve epileptiform discharge detection.

**Methods:**

Retrospective analysis of patients who underwent continuous EEG monitoring with pharmacologic paralysis. Four reviewers read each patient’s EEG pre- and post- both paralysis and AR, and indicated the presence of epileptiform discharges. We compared the interrater reliability (IRR) of identifying discharges at baseline, post-AR, and post-paralysis, and compared the performance of AR and paralysis according to artifact type.

**Results:**

IRR of identifying epileptiform discharges at baseline was slight (*N* = 30; κ = 0.10) with a trend toward increase post-AR (κ = 0.26, *p* = 0.053) and a significant increase post-paralysis (κ = 0.51, *p* = 0.001). AR was as effective as paralysis at improving IRR of identifying discharges in those with high EMG artifact (*N* = 15; post-AR κ = 0.63, *p* = 0.009; post-paralysis κ = 0.62, *p* = 0.006) but not with primarily myoclonus artifact (*N* = 15).

**Conclusions:**

Paralysis improves detection of epileptiform activity in critically ill patients when movement-related artifact obscures EEG features. AR improves detection as much as paralysis when EMG artifact is high, but is ineffective when the primary source of artifact is myoclonus.

**Significance:**

In the appropriate setting, both AR and paralysis facilitate identification of epileptiform activity in critically ill patients.

## 1. Introduction

Non-convulsive seizures are common in critically ill patients, and carry important treatment and prognostic implications (Hill et al., 2019; Oddo et al., 2009; Schmitt, 2017). Therefore, critically ill patients are commonly monitored on continuous electroencephalography (EEG) if they are thought to be at high risk of seizures or if they have abnormal movements concerning for seizures. In such patients, recognizing epileptiform discharges that correlate with concerning movements is thought to be diagnostic of electroclinical seizures (Hirsch et al., 2021).

Movement-related artifacts often obscure EEG recordings and limit the detection of epileptiform discharges. Two common types of movement-related artifact in critically ill patients are electromyography (EMG) artifact and myoclonus artifact. EMG artifact is high frequency, and is often continuous and high amplitude in the setting of rigors, tremors, and stiffening. Myoclonus artifact correlates to clinical myoclonus, which can occur in a variety of muscle groups and leads to brief, paroxysmal (though sometimes quite frequent), high amplitude artifacts on EEG.

Multiple approaches exist to reducing movement-related artifacts. Pharmacologic paralysis inhibits skeletal muscle activity and can eliminate movement-related artifacts. Paralysis is used selectively at our institution and others to elucidate the underlying cerebral waveforms in intubated patients with excessive movement artifact on EEG (Newey et al., 2017; Schmitt, 2018; White and Van Cott, 2010; Young and Mantia, 2017). However, non-depolarizing neuromuscular blockers are associated with side effects including histamine-mediated tachycardia, hypotension, bronchospasm, and urticaria, as well as residual neuromuscular blockade, especially in patients with various neurological comorbidities including myasthenia gravis, which could potentially prolong the need for mechanical ventilation (Clar and Liu, 2021; Debaene et al., 2003). Commercially-available software now exists for quantitative artifact reduction (AR), which uses blind source separation methods to identify and remove EEG components likely to be artifacts (Fitzgibbon et al., 2016, 2007; Islam et al., 2016; Janani et al., 2020, 2018; Urigüen and Garcia-Zapirain, 2015; Whitham et al., 2007). An important advantage of quantitative AR is that it can be performed both retrospectively and in realtime on EEG without requiring any special data collection or clinical intervention beyond the EEG itself (Artifact Reduction, 2022). The ability of pharmacologic paralysis and quantitative AR to improve detection of epileptiform discharges in critically ill patients is unknown.

In this study, we tested the ability of both pharmacologic paralysis and quantitative AR to improve detection of epileptiform discharges in critically ill patients. We performed a retrospective analysis of critically ill patients who underwent continuous EEG and had pharmacologic paralysis for the purpose of detecting epileptiform discharges. We compared the interrater reliability (IRR) of epileptiform discharge detection in three conditions: baseline, post-AR, and post-paralysis. We hypothesized that both paralysis and quantitative AR would improve the IRR of the detection of epileptiform discharges.

## 2. Methods

### 2.1. Study design and participants

This was a retrospective study of intensive care unit inpatients throughout the University of Pennsylvania hospital system monitored on continuous EEG between 2018 and 2021. Inclusion criteria required patients to be (1) intubated in the intensive care unit, with (2) clinical concern for seizures, requiring (3) pharmacological paralysis to make this determination. Patients were excluded if paralytics were not required for EEG interpretation (e.g., administered solely for medical management or targeted temperature management protocol).

### 2.2. Data collection and management

A custom EEG database (developed by J.P.) was queried for the term paraly* (^“*”^ is a wildcard marker) between 2018–2021. All EEG reports were reviewed manually, and additional chart review was also undertaken if needed for clarification. Demographic information, clinical history, medications, myoclonus semiology, and EEG data were collected by chart review.

Continuous video EEGs were clipped to create separate files containing the EEG tracings during the 4 hours preceding (or from initial connection, if less than 4 hours) and the 4 hours following the administration of a paralytic, excluding the 10 minutes after receiving paralytic. Paralysis was confirmed by visual inspection of the EEG showing resolution of EMG and myoclonus artifact. Train-of-four was not used consistently for every patient and therefore was not a criterion to determine paralysis in this retrospective study. All pre- and post-paralysis EEG clips were de-identified from the patient and given a unique numerical identifier. The corresponding video files were no longer available at the time of this study. Per chart review and prior clinical EEG annotations, study clips were labeled with the presence or absence of myoclonus and the semiology, if applicable.

**Fig. 1.**
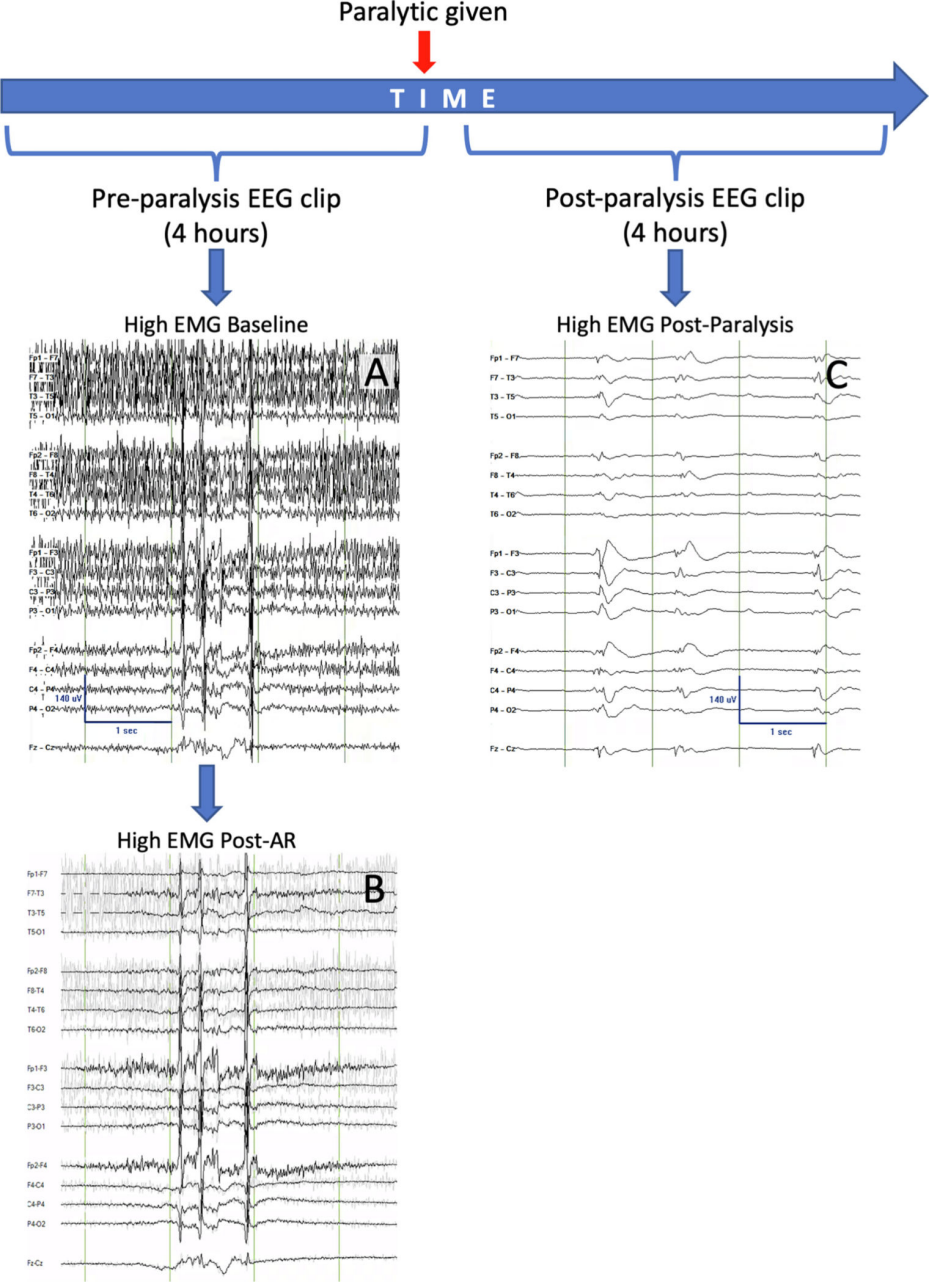
Schematic of study design. Visual representation of the timing and source of baseline (A), post-artifact reduction (AR; B), and post-paralysis (C) EEG clips.

**Fig. 2.**
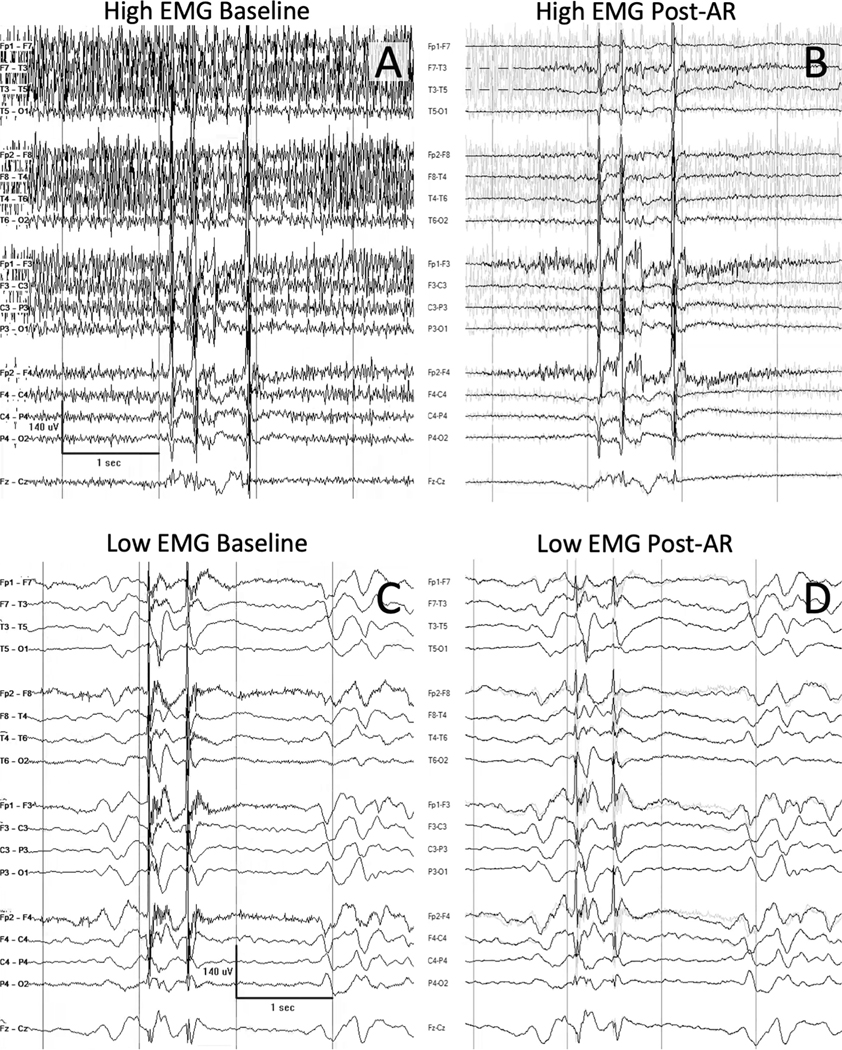
Representative baseline and post-artifact reduction (AR) EEG tracings for patients with high and low EMG artifact. Representative images for a patient with high EMG artifact on EEG at baseline (A) and after AR (B), compared to a patient with low EMG artifact at baseline (C) and after AR (D).

Fig. 1 shows a schematic of the study design. EEG clips were reviewed by four board-certified epileptologists (E.C, T.G., R.R., C. E.). The amount of EMG artifact (high vs low), the presence of myoclonus artifact, the presence of convincing epileptiform discharges, and the confidence in EEG interpretation (high vs low) were recorded for each EEG clip by each reviewer. EEG reviewers were instructed to record their baseline pre-paralysis responses prior to applying AR, and then review the EEG again using the Persyst 13 AR tool (Persyst Development Corporation, California, USA; Fig. 2). Reviewers were instructed not to change their baseline pre-paralysis responses after applying AR. Reviewers were blinded as to which pre-paralysis EEG corresponded to which post-paralysis EEG.

Pre-paralysis EEGs were classified as having high versus low EMG artifact based on majority consensus (E.C, T.G., R.R., C.E.). Four cases in which there was no consensus were reviewed and adjudicated by a board-certified epileptologist (E.C.). The final post-paralysis clinical determination of whether epileptiform discharges were present (as indicated in the post-paralysis clinical EEG report) was noted. All study data were collected and stored using REDCap (Research Electronic Data Capture) tools (Harris et al., 2019, 2009).

### 2.3. Data analysis

For our primary analysis, we measured the IRR between different reviewers’ EEG interpretations (presence or absence of epileptiform discharges) for each of the three conditions: baseline pre-paralysis, post-AR pre-paralysis, and post-paralysis. Fleiss’ kappa was used to measure IRR, which provides a single measure of agreement for multiple raters, compared to chance (κ = 0). IRR was chosen as our primary accuracy measure given its prior use in studies of EEG interpretation (Grant et al., 2014; Newey et al., 2017). As a secondary analysis of EEG interpretation, we also compared the agreement between the reviewers’ interpretation and the final post-paralysis clinical determination of whether epileptiform discharges were present. Agreement was defined as the number of EEGs in which the reviewer had the same interpretation as the clinical report divided by the total number of EEGs. This was averaged amongst all reviewers for each condition. Confidence was defined as the number of EEGs in which the reviewer marked that they were confident in their interpretation, again averaged amongst all reviewers for each condition.

Finally, we also measured average reviewer sensitivity and specificity for detection of epileptiform discharges relative to the clinical report. Sensitivity was defined as the number of true positives (EEGs with epileptiform discharges according to both the reviewer and the clinical report) divided by the total number of EEGs with epileptiform discharges according to the post-paralysis clinical report. Specificity was defined as the number of true negatives (EEGs without epileptiform discharges according to both the reviewer and the clinical report) divided by the total number of EEGs without epileptiform discharges according to the post-paralysis clinical report.

We compared the IRR, the agreement with the post-paralysis clinical report, the sensitivity, the specificity, and the confidence between the baseline and post-AR conditions, and between the baseline and post-paralysis conditions. We tested for significant differences between the conditions using clustered bootstrapping (Field and Welsh, 2007). In this approach, 30 EEGs were randomly sampled from the original set of 30 EEGs with replacement. IRR, average agreement with the clinical report, average confidence, sensitivity, and specificity were re-calculated for each randomly sampled set of EEGs. The difference between the baseline statistic (IRR, average agreement, average confidence, sensitivity, or specificity) and the post-AR or post-paralysis statistic was obtained. Resampling was performed 10,000 times to obtain a distribution of statistic differences. The one-sided *p*-value was defined as the proportion of statistic differences less than or equal to zero (Vanbelle and Albert, 2008). We performed clustered bootstrapping because we expected the reviewer interpretations to be highly correlated within EEGs. This would lead to higher variances than would be expected if each EEG review were treated independently, and thus ignoring this clustering would increase the probability of Type I errors. Finally, we compared the odds of survival to discharge amongst patients who were diagnosed with epileptiform discharges and those without using a Fisher’s exact test.

### 2.4. Standard protocol approvals, registrations, and patient consents

This study was approved by the institutional review board (IRB) at the University of Pennsylvania. Written informed consent was waived by the IRB to avoid storing identifying data.

### 2.5. Data and code availability

De-identified data and code to perform the analysis is available on https://github.com/erinconrad/paralysis/.

## 3. Results

### 3.1. Demographic and clinical data

Of the 30 patients who met inclusion criteria, the mean age was 63 (range 36–90), and 20 patients (67 %) were male (Table 1). Most (*N* = 26, 87 %) did not have a history of epilepsy. The inciting event for 25 patients (83 %) was cardiac arrest, for 2 (7 %) was severe shock, and for 3 (10 %) was acute encephalopathy. All were sedated with some combination of fentanyl, propofol, ASMs, and/or benzodiazepines. Patients were paralyzed with either cisatracurium or vecuronium. In all cases, paralytics were clinically required for EEG interpretation; in most cases (*N* = 28, 93 %) this was in order to distinguish between epileptic and non-epileptic myoclonus; the other two patients were paralyzed due to the presence of severe continuous EMG artifact precluding EEG interpretation. The average number of days between the inciting event and administration of a pharmacological paralytic for EEG interpretation was 2 (SD 4, range 0–13). Most patients (*N* = 24, 80 %) were paralyzed within 72 hours. According to the final post-paralysis clinical report, 17 patients (57 %) were ultimately determined to have epileptiform discharges. Incidentally, every patient that was clinically determined to have epileptiform discharges also had myoclonus as a clinical correlate, and thus met American Clinical Neurophysiology Society (ACNS) criteria for electroclinical seizures (Hirsch et al., 2021).

### 3.2. Improvement in detection of epileptiform discharges after quantitative AR and pharmacologic paralysis

**Fig. 3.**
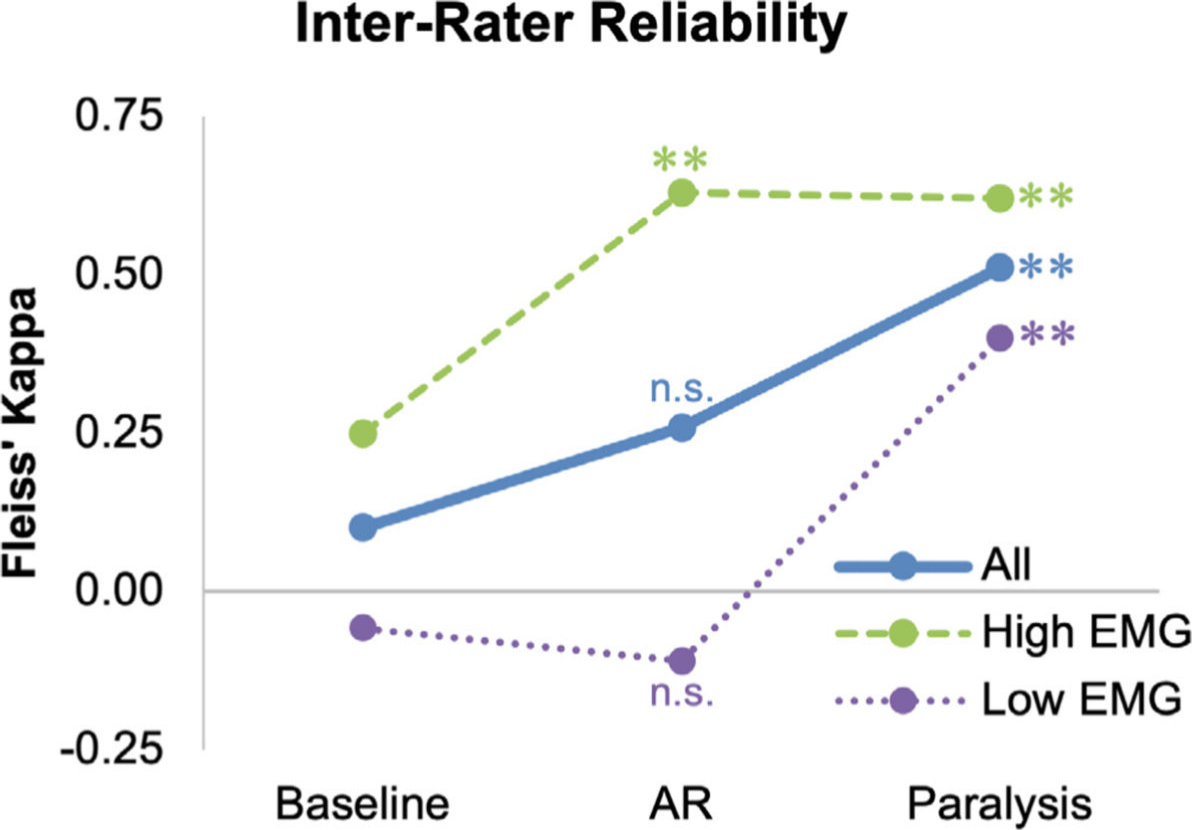
Interrater reliability (IRR) of baseline, post-artifact reduction (AR), and post-paralysis EEG reads. IRR across multiple conditions. The x-axis indicates the condition (baseline, post-AR, or post-paralysis) and the y-axis shows the IRR as measured by Fleiss’ kappa. The solid line shows the primary analysis of all patients (“All”), the dashed line shows the analysis for patients with high EMG artifact (“High EMG”), and the dotted line shows the analysis for patients with low EMG artifact (“Low EMG”). For the primary analysis, there was a non-significant trend toward improvement in IRR with AR and significant improvement after paralysis. For patients with high EMG artifact, IRR similarly and significantly improved with both AR and paralysis. For patients with low EMG artifact, IRR did not improve after AR but significantly improved after paralysis. The asterisks represent the statistical significance of the difference between a given condition and the baseline condition (hence there is no significance marker above the baseline condition because this is the comparison group). ** = p ≤ 0.01 when compared to baseline, n.s. = not significant.

**Fig. 4.**
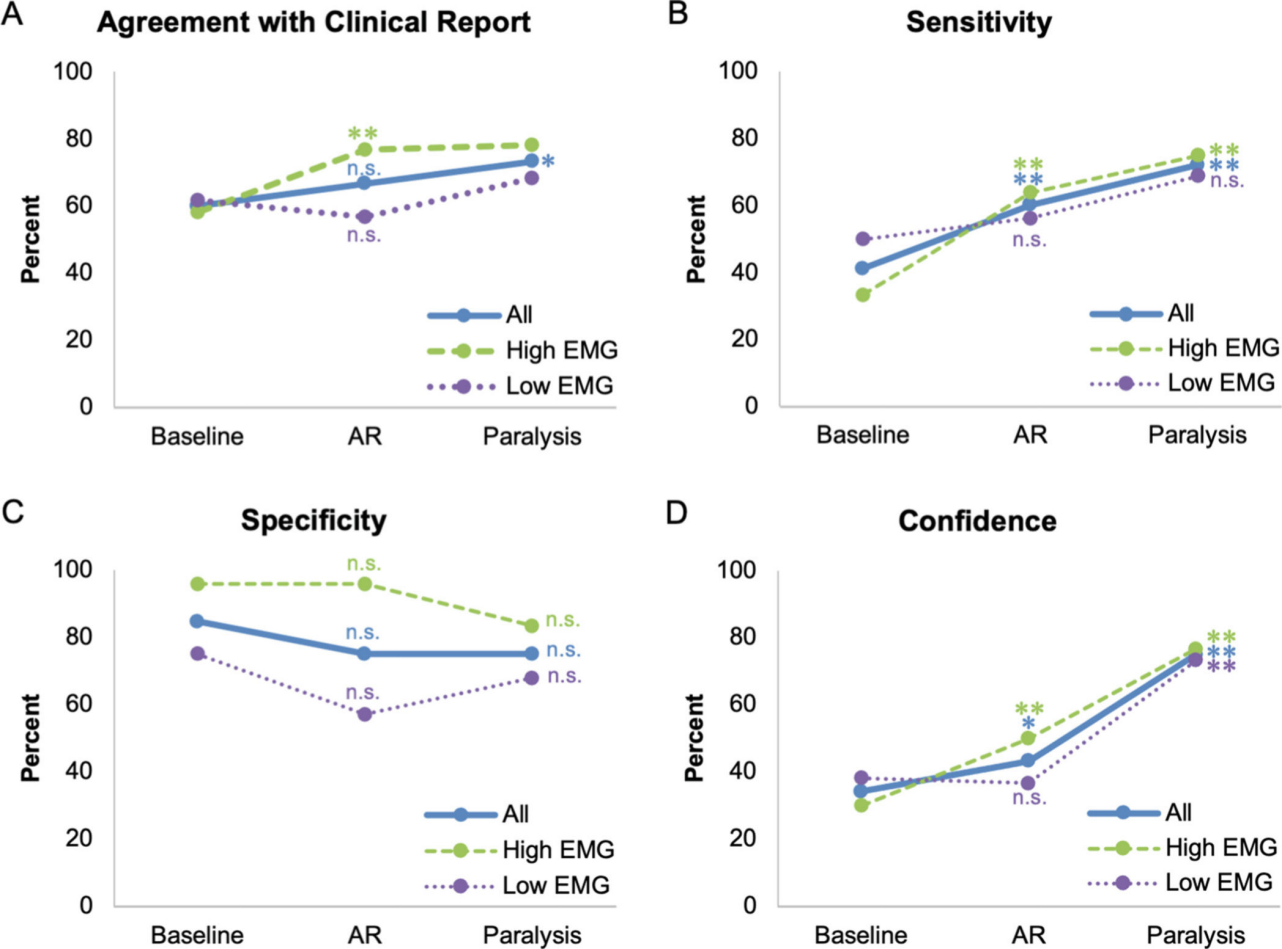
Agreement with clinical report, sensitivity, specificity, and confidence of baseline, post-artifact reduction (AR), and post-paralysis EEG reads. Performance of reviewers at detecting epileptiform discharges across different artifact reduction interventions, as measured by (A) percent agreement with clinical report, (B) sensitivity compared to clinical report, (C) specificity compared to clinical report, (D) reviewer confidence in their interpretation. In each plot, the solid line shows the primary analysis of all patients (*N* = 30), the dashed line shows the analysis for patients with high EMG artifact (*N* = 15), and the dotted line shows the analysis for patients with low EMG artifact (*N* = 15). Stars represent statistical significance of each datapoint compared to the baseline measure of the same condition. * = p ≤ 0.05 when compared to baseline, ^**^ = p 0.01 when compared to baseline, n.s. = not significant.

Table 2 and Figs. 3 and 4 show a summary of our results. Table 3 shows the strength of agreement using Fleiss’ kappa (κ). Note that chance agreement for IRR regarding presence/absence of epileptiform discharges using Fleiss’ kappa (Fig. 3) would be κ = 0 whereas chance agreement between reviewer interpretation and the clinical report regarding presence/absence of epileptiform discharges (Fig. 4) would be 50 %. At baseline (unparalyzed EEG segments), the IRR of the four epileptologists’ EEG reads was only slight (κ = 0.10, Table 2, Table 3, Fig. 3)(Grant et al., 2014; Landis and Koch, 1977). IRR improved to fair after AR (κ = 0.26, 95 % CI for the difference in κ between AR and baseline [−0.03, 0.36], *p* = 0.053), and to moderate after paralysis (κ = 0.51, 95 % CI for the difference in κ between paralysis and baseline [0.18, 0.64], *p* = 0.001). The average agreement between the reviewer interpretation and the clinical report was 60.0 % at baseline (Fig. 4). Agreement with the clinical report increased to 66.7 % after AR (*p* = 0.08), and 73.3 % after paralysis (*p* = 0.050). The sensitivity of detecting convincing epileptiform discharges was only 41.2 % at baseline, but significantly increased to 60.3 % (*p* < 0.001) after AR and 72.1 % (*p* < 0.001) after paralysis. The specificity of detecting convincing epileptiform discharges was high at baseline (84.6 %), and remained so after AR (75.0 %, *p* = 0.98) and after paralysis (75.0 %, *p* = 0.84). The average percent of EEG interpretations that were identified as highly confident was low at baseline (34.2 %), but significantly increased after AR (43.3 %, *p* = 0.044) and after paralysis (75.0 %, *p* < 0.001).

### 3.3. Subgroup analysis of high EMG subset

The 30 pre-paralysis EEG clips were divided into two subsets of high EMG artifact (15 patients, Fig. 2A–B) and low EMG artifact (15 patients, Fig. 2C–D) based on majority consensus, as outlined above. The IRR of the high EMG group was only fair at baseline (κ = 0.25, Table 2, Table 3, Fig. 3), but significantly increased to substantial after AR (κ = 0.63, 95 % CI for the difference in κ between AR and baseline [0.06, 0.69], *p* = 0.009) and paralysis (κ = 0.62, 95 % CI for the difference in κ between paralysis and baseline [0.08, 0.73], *p* = 0.006). The agreement of the EEG reads with the clinical report in this group was 58.3 % at baseline and increased to 76.7 % (*p* = 0.002) after AR and 78.3 % (*p* = 0.056) after paralysis (Fig. 4). The higher p-value in the baseline-to-paralysis change compared to the baseline-to-AR change (disagreeing with intuition) largely reflects the fact that changes in agreement were more heavily clustered amongst EEGs in the former condition (for example, for three of the 15 EEGs, the interpretation of the presence or absence of epileptiform discharges switched for all four reviewers going from baseline-to-paralysis; no similar clustering was observed in the baseline-to-AR condition). Thus, when performing clustered bootstrapping, the improvement in agreement was more heavily dependent on which EEGs were randomly chosen in the baseline-to-paralysis analysis than in the baseline-to-AR analysis, implying higher variance in the baseline-to-paralysis analysis when performing clustered bootstrapping. The sensitivity of detecting convincing epileptiform discharges in the high EMG group was only 33.3 % at baseline, but significantly increased to 63.9 % (*p* < 0.001) after AR and 75.0 % (*p* < 0.001) after paralysis. The specificity of detecting convincing epileptiform discharges was high at baseline (95.8 %), and remained so after AR (95.8 %, *p* = 0.65) and after paralysis (83.3 %, *p* = 0.76). The average percent of interpretations that were identified as highly confident was low at baseline (30.0 %), but significantly increased after AR (50.0 %, *p* = 0.009) and after paralysis (76.7 %, *p* < 0.001).

### 3.4. Subgroup analysis of low EMG subset

For patients with low EMG artifact, the primary source of artifact was myoclonus. The IRR of the low EMG group was poor at baseline (κ = −0.06) and did not increase after AR (κ = −0.11, 95 % CI for the difference in κ between AR and baseline [−0.24, 0.11], *p* = 0.71), but did increase to fair after paralysis (κ = 0.40, 95 % CI for the difference in κ between paralysis and baseline [0.09, 0.81], *p* = 0.009). Agreement of the EEG reads with the clinical report in this group was 61.7 % at baseline, and did not significantly improve after AR (56.7 %, *p* = 0.89) or paralysis (68.3 %, *p* = 0.27). There was no significant change in sensitivity (50.0 % baseline; 56.2 % AR, *p* = 0.12; 68.8 % paralysis, *p* = 0.08) or specificity (75.0 % baseline; 57.1 % AR, *p* = 1.00; 67.9 % paralysis, *p* = 0.74) across conditions. Confidence did not increase after AR (36.7, *p* = 0.70), but it did increase after paralysis (38.3 % to 73.3 %, *p* < 0.001).

### 3.5. Clinical outcome

Of the 30 patients included in the study, 9 (30.0 %) survived to hospital discharge. This did not differ based on whether the patient did (29.4 %) or did not (30.8 %) have epileptiform discharges on EEG (odds ratio 0.94, 95 % CI [0.19, 4.52], *p* = 1.00).

## 4. Discussion

### 4.1. Overview

We analyzed EEGs of 30 critically ill patients who underwent continuous EEG recording and had pharmacologic paralysis for the purpose of identifying epileptiform discharges. We compared the IRR for the detection of epileptiform discharges between EEGs at baseline and post-AR, and between EEGs at baseline and post-paralysis. We found that paralysis improves the IRR of detecting epileptiform discharges regardless of artifact type. On the other hand, AR only improves EEG interpretation in patients with high EMG artifact, but not those with low EMG artifact. In the setting of high EMG artifact, AR is as effective as pharmacologic paralysis at improving EEG interpretation. Overall, our results suggest that both pharmacologic paralysis and quantitative AR can improve the detection of epileptiform activity in appropriately chosen critically ill patients.

### 4.2. Patients requiring pharmacologic paralysis for EEG interpretation are frequently having seizures

In our study, roughly half (53 %) of the patients were ultimately found to have epileptiform discharges and thus, in the setting of myoclonus, met ACNS criteria for electroclinical seizures (Hirsch et al., 2021). This proportion is higher than that reported in the literature for critically ill patients undergoing continuous EEG more generally (10–30 %)(Oddo et al., 2009; Schmitt, 2017). This may be because our patients had clinical presentations suggesting a higher risk of seizures: most were post-cardiac arrest, and most had myoclonus. Given the high pretest probability of seizure in these patients, clinicians might consider empirically treating post-arrest patients with myoclonus prior to performing paralysis or other interventions to identify epileptiform discharges in the context of EEGs with substantial movement artifact. Also, the relatively even split between patients who were and were not having seizures highlights the need for methods to better identify epileptiform activity, including non-convulsive seizures, to guide therapy. At baseline, the IRR of EEG interpretation in these patients was only marginally better than chance (κ = 0.10), further supporting the need for improved diagnostic methods.

### 4.3. Paralysis improves EEG interpretation in the setting of both types of movement artifact

As anticipated, paralysis increased the IRR of EEG reads. This was true when studying all patients, as well as in both subgroups of EEGs with high EMG artifact and those with low EMG artifact. The IRR in the post-paralysis setting was κ = 0.51, considered “moderate agreement” (Grant et al., 2014; Landis and Koch, 1977). For comparison, a separate study of the IRR of scalp EEG interpretation more generally (identifying a range of abnormal EEG features) found an average κ = 0.44 when seven EEG categories were used and κ = 0.55 when three EEG categories were used (Grant et al., 2014). Our similar IRR even post-paralysis highlights the persistent diagnostic challenge of identifying epileptiform abnormalities in the critically ill population. Regardless, the substantial improvement in IRR with paralysis (κ = 0.10 → 0.51) implies that paralysis is a useful diagnostic tool. The diagnostic utility of pharmacologic paralysis must be weighed against its associated risks (Clar and Liu, 2021; Debaene et al., 2003).

### 4.4. Quantitative AR improves EEG interpretation in the setting of high EMG artifact, but not in the setting of primarily myoclonus artifact

In aggregate, there was a non-significant trend toward improved IRR of EEG interpretation after applying AR. When we separated EEGs according to the amount of EMG artifact present, IRR significantly increased after applying AR in the high EMG group, but not the low EMG group. This aligns with reviewers’ greater confidence gains pre-to-post AR in the high EMG group than in the low EMG group. Visually, we found that after applying AR to high EMG clips, the morphology of any remaining epileptiform discharges appeared to be preserved, and matched the post-paralysis morphology (Fig. 2A–B). In contrast, applying AR to clips that were primarily obscured by myoclonus artifact often did not adequately visually suppress the myoclonus artifact. The different performance in AR for EEGs with high EMG artifact and those with low EMG artifact agrees with the product literature for Persyst Artifact Reduction, which states that AR reduces EMG, electrode, and eye movement artifact (Artifact Reduction, 2022; Nierenberg et al., 2013).

Most patients in our study (28 out of 30) had myoclonus, and thus we could not meaningfully compare the performance of AR methods in the presence versus the absence of myoclonus. The fact that quantitative AR was effective in the high EMG group despite the fact that most of these patients also had myoclonus suggests that, for these patients, it was the more continuous EMG artifact rather than the myoclonus that primarily obscured EEG interpretation.

In the high EMG group, the magnitude of the improvement in IRR after applying AR was comparable to that achieved by paralysis (AR κ = 0.63, paralysis κ = 0.62, both considered “substantial agreement”). This result suggests that in critically ill patients for whom EMG artifact obscures EEG interpretation, paralysis may be unnecessary, as quantitative AR achieves similar (and good) accuracy. However, in the setting of low EMG (with high myoclonus artifact), AR is unlikely to be helpful, and paralysis may be necessary for diagnosis.

### 4.5. Limitations

A relatively small number of patients (*N* = 30) met the study criteria. Our patient population was primarily post-cardiac arrest with clinical myoclonus, so caution should be used when applying these findings to critically ill patients with other clinical contexts. Also, EEG readers did not have access to video files from the video EEGs, and therefore we could not fully replicate the typical conditions of a clinical reviewer. This may have been helpful to elucidate semiology and to determine if EEG waveforms aligned with clinical myoclonus. However, in each clinical case the myoclonus was unable to be categorized as epileptic or non-epileptic in real time based on the unparalyzed EEG tracing and video alone, so we do not believe that the video would resolve the diagnostic dilemma in the study. Additionally, myoclonus was often intermittent rather than continuous, raising the possibility that the episodes of interest may not have been captured within the pre- or post-paralysis EEG. For this reason we used prolonged (4 hour) EEG clips whenever possible, as opposed to the usual routine EEG length (20 minutes). Given that the post-AR reviewer interpretation was based on the EEG pattern at a different timepoint than the post-paralysis reviewer interpretation and post-paralysis clinical report, the agreement with the post-paralysis clinical report, the sensitivity, and the specificity of the post-AR reads may be falsely lowered compared to the post-paralysis reads. Also, the use of the post-paralysis clinical report as our “gold standard” in measuring agreement, sensitivity, and specificity is limited by the data, in our study and in others (Grant et al., 2014), that inter-rater reliability of EEG interpretation is imperfect (and thus the clinical report by a single epileptologist may be flawed). Lastly, it should be noted that the results of this study are based on the performance of only one commercially available software, and future updates or alternative algorithms may perform differently.

### 4.6. Clinical applicability

The clinical significance of identifying and treating epileptiform activity in certain critically ill populations remains a topic of debate. A large retrospective study found that the use of continuous EEG is associated with improved outcomes for critically ill patients, suggesting that identifying and treating seizures and other epileptiform patterns is helpful in these patients (Hill et al., 2019). On the other hand, the recent large prospective TELSTAR trial found no difference in outcome for post-cardiac arrest patients who received standard of care versus targeted therapy to treat epileptiform EEG patterns (Ruijter et al., 2022). The literature on post-arrest outcomes is limited more generally by the fact that most of these patients who die do so from withdrawal of care, reducing the power to find a difference in long-term outcome amongst those in whom care is not withdrawn. In our study, survival to hospital discharge was 30 %, and did not differ between those with and those without epileptiform discharges. Long-term outcome data is not available for most patients, and our study is likely underpowered to detect a difference in outcome given the size of our study compared to the outcome studies cited above. Nevertheless, when EEG recording is performed in these patients with the goal of detecting epileptiform patterns, there is a need to optimize that detection in the face of common sources of artifact in the ICU environment.

## 5. Conclusion

In summary, we found that paralysis is effective for improving the detection of epileptiform discharges in critically ill patients with movement-related artifacts obscuring EEG interpretation. Quantitative AR is effective only in patients whose EEGs are obscured by high EMG artifact, but not those with low EMG artifact. Amongst patients with high EMG artifact, AR is as effective as paralysis at improving detection of epileptiform discharges. Therefore, in critically ill patients with suspected seizures whose EEGs are obscured by high EMG artifact, it may be reasonable to apply quantitative AR—without paralysis—to determine the presence or absence of epileptiform discharges. However, in patients with low EMG artifact but predominant myoclonus artifact, pharmacologic paralysis may be necessary for diagnosis.

## Figures and Tables

**Table 1:** Demographic and clinical data. Basic demographic and clinical data for the cohort of patients included in this study. “Interval days” refers to the number of days between the inciting event and administration pf paralysis. Epileptiform discharges refers to the final clinical report, including post-paralysis recording.

Variable	All subjects N = 30
**Age** Mean (SD, range)	63 (13, 36–90)
**Sex** N (%)	
Male	20 (67 %)
Female	10 (33 %)
**History of epilepsy** N (%)	
Yes	4 (13 %)
No	26 (87 %)
**Event** N (%)	
Cardiac arrest	25 (83 %)
Shock / peri-arrest	2 (7 %)
Encephalopathy	3 (10 %)
**Interval days** Mean (SD, range)	2 (4, 0–13)
**Clinical myoclonus** N (%)	
Yes	28 (93 %)
No	2 (7 %)
**Epileptiform discharges** N (%)	
Yes	17 (57 %)
No	13 (43 %)
**Mortality** N (%)	
Survived to discharge	9 (30 %)

N = number of patients. SD = standard deviation.

**Table 2:** Comparison of baseline, artifact reduction, and paralysis EEG reads. Inter-rater reliability (κ, Fleiss’ kappa) of EEG reads, agreement between EEG reads and the clinical report, sensitivity and specificity of identifying epileptiform discharges, and confidence in EEG reads at baseline, after AR, and after paralysis. This was determined for all subjects as well as for the subset of EEG tracings with high EMG artifact and low EMG artifact.

	All subjects N = 30			High EMG N = 15			Low EMG N = 15		
			
Variable	Baseline	AR	Paralysis	Baseline	AR	Paralysis	Baseline	AR	Paralysis
**Inter-rater reliability** (κ)	0.10	0.26	**0.51** ^**^	0.25	**0.63** ^**^	**0.62** ^**^	−0.06	−0.11	**0.40** ^**^
**Agreement with report** (%)	60.0	66.7	**73.3** ^*^	58.3	**76.7** ^**^	78.3	61.7	56.7	68.3
**Sensitivity** (%)	41.2	**60.3** ^**^	**72.1** ^**^	33.3	**63.9** ^**^	**75.0** ^**^	50.0	56.2	68.8
**Specificity** (%)	84.6	75.0	75.0	95.8	95.8	83.3	75.0	57.1	67.9
**High confidence** (%)	34.2	**43.3** ^*^	**75.0** ^**^	30.0	**50.0** ^**^	**76.7** ^**^	38.3	36.7	**73.3** ^**^

N = number of patients. AR = artifact reduction.

* = p ≤ 0.05 when compared to baseline

** = p ≤ 0.01 when compared to baseline.

**Table 3:** Kappa statistic. Strength of agreement using Fleiss’ kappa (κ)(Grant et al., 2014; Landis and Koch, 1977).

κ Value	Strength of Agreement
< 0	Poor
0 – 0.20	Slight
0.21 – 0.40	Fair
0.41 – 0.60	Moderate
0.61 – 0.80	Substantial
0.81 – 1.00	Almost perfect

## HIGHLIGHTS

- Pharmacologic paralysis improves detection of EEG epileptiform activity regardless of type of movement-related artifact present.
- Artifact reduction improves detection of EEG epileptiform activity as much as pharmacologic paralysis when primary source of artifact is EMG.
- In the appropriate setting, both artifact reduction and paralysis facilitate identification of epileptiform activity in critically ill patients.
